# Fibrous Structures Produced Using the Solution Blow-Spinning Technique for Advanced Air Filtration Process

**DOI:** 10.3390/ma16227118

**Published:** 2023-11-10

**Authors:** Agata Penconek, Anna Jackiewicz-Zagórska, Rafał Przekop, Arkadiusz Moskal

**Affiliations:** Faculty of Chemical and Process Engineering, Warsaw University of Technology, 00-645 Warsaw, Poland; anna.jackiewicz@pw.edu.pl (A.J.-Z.); rafal.przekop@pw.edu.pl (R.P.)

**Keywords:** non-woven filter, solution blow spinning, PLA, air cleaning

## Abstract

This study proposes utilising the solution blow-spinning process (SBS) for manufacturing a biodegradable filtration structure that ensures high efficiency of particle filtration with an acceptable pressure drop. The concept of multi-layer filters was applied during the design of filters. Polylactic acid (PLA) was used to produce various layers, which may be mixed in different sequences, building structures with varying filtration properties. Changing the process parameters, one can create layers with diverse average fibre diameters and thicknesses. It enables the design and creation of optimal filtration materials prepared for aerosol particle filtration. The structures were numerically modelled using the lattice Boltzmann approach to obtain detailed production guidelines using the blow-spinning technique. The advantage of this method is the ability to blow fibres with diameters in the nanoscale, applying relatively simple and cost-effective equipment. For tested PLA solutions, i.e., 6% and 10%, the mean fibre diameter decreases as the concentration decreases. Therefore, the overall filtering efficiency decreases as the concentration of the used solution increases. The produced multi-layer filters have 96% overall filtration efficiency for particles ranging from 0.26 to 16.60 micrometres with a pressure drop of less than 160 Pa. Obtained results are auspicious and are a step in producing efficient, biodegradable air filters.

## 1. Introduction

With the development of civilisation, the awareness of the impact of versatile aerosol particles on human health and the environment is growing. It is estimated that approximately 3% of deaths caused by circulatory-respiratory failure and 5% of lung cancer are the result of exposure to particulate matter [1]. When the concentration of PM10 increases by 10 μg/m^3^, the daily mortality increases by 0.6% daily, chronic obstructive pulmonary disease (COPD) in people over 65 increases by 10% and the risk of heart disease increases by 11% [2]. Exposure to particulate matter also impairs lung development in children, leading to chronic dysfunction [1]. To reduce this impact, new advanced materials are needed to purify the air efficiently during filtration. First, new materials should have a small environmental impact due to the availability of the recycling process or the ability to biodegrade. Secondly, optimising the fibrous structure’s morphology can reduce energy (due to a decrease in the pressure drop) with the same or higher filtration efficiency. There are many ways to reduce the aerosol particles generated by both industry and automobiles. However, most of them have a limited collection efficiency for particles with diameters below 2 μm [3]. The efficiency of airborne particle separation drastically drops for particles with diameters less than 2 μm, which means that the particles most dangerous for human health (respirable fraction) are kept to a very limited extent. Conventional dust captures devices such as electrostatic precipitators, cyclones, and wash towers do not appropriately meet the high standards, and they are far less efficient in collecting sub-micron particles. A non-woven filter structure can provide effective separation. There are three main methods of producing non-woven filter structures: melt blow, electrospinning and solution blow spinning. The most economically advantageous method (due to the lack of solvent) is melt blow. The average diameters of the obtained fibres are in the range of 1–50 µm. However, the process is limited to heat-resistant polymers; therefore, the most commonly used polymers are polypropylene and polyethylene. Electrospinning, in turn, allows obtaining fibres in the nanometre range, but the efficiency of the process is low, and the use of high voltage is additionally required. However, electrospinning allows the production of fibres from polymers such as silk, corn, soy, collagen, and gluten [4]. The solution blow-spinning technique allows the obtaining of fibres and nanofibers that can be used in filtration structures with designed morphology (average fibre diameter, structure thickness, porosity and fibre arrangement) without using high voltage. Fibres obtained in solution blow spinning can also be used in, for example, biomedical applications such as wounds [5] or scaffolds [6].

The formation of fibres in the solution blow spinning is detailed in the literature, e.g., [7,8,9,10,11]. The process was first described in 2009 by Medeiros et al. [12]. The polymer flows out of the nozzle and forms a droplet. The droplet changes its shape into a cone due to shear stresses generated by the air flowing around the nozzle. The air velocity at the nozzle outlet is high due to its expansion to atmospheric pressure. A polymer jet is formed from the cone under stretching and shearing forces. After the solvent evaporation, the fibre is created and deposited on a collector. The collectors have various shapes and can also rotate. In the case of cylindrical collectors that rotate along their long axis, the deposited fibres’ alignment increases with the rotational speed. However, as the distance between the collector and the nozzle decreases, the efficiency of fibre capture increases. The ability to control fibre capture is crucial, as in solution blow spinning, nanofibers are produced (similar to electrospinning) [13]. The presence of nanofibers in the structure increases the specific surface area, which is advantageous for capturing and trapping airborne particles. However, the efficiency of fibre capture decreases as their diameter decreases. An essential feature of solution blow spinning is the numerous process parameters that can be controlled to configure the properties of the filtration structure. In addition to the parameters mentioned earlier, the polymer flow rate, airflow rate, polymer concentration, nozzle diameter, temperature and air humidity are also controlled.

The average fibre diameter increases with an increase in the polymer flow rate from the nozzle [14]. Still, excessive flow rates may lead to issues with solvent evaporation and fibre formation [15]. The increased airflow rate reduces fibre diameter and elongation due to the increased stretching force acting on the extruded polymer jet. The fibre diameter and airflow velocity relationship sometimes follow a parabolic shape [12,16]. Conversely, an increase in polymer concentration, due to the increased apparent viscosity of the solution and surface tension, increases fibre thickness [14].

As the nozzle diameter decreases, the average fibre diameter should increase due to greater internal friction between the solution and the inner surface of the nozzle [17]. However, a large nozzle diameter can form large, fused fibres because the solvent does not have sufficient time to evaporate from the polymer jet [15]. Temperature and air humidity affect the solvent evaporation rate from the polymer jet. The influence of temperature and humidity is closely related to the type of polymer and solvents used.

The multiparametric nature of solution blow spinning allows it to successfully create multi-layer structures, where different fibrous layers have varying properties, resulting in varying filtration efficiency and pressure drop across the layers.

Using different polymers is crucial from the perspective of future applications of the manufactured filtration structures. The most popular polymers used in polymer solution blowing are biodegradable PLA (polylactic acid); PLGA (poly(lactic-co-glycolic acid)); PCL (poly-ε-caprolactone), e.g., [18,19,20,21]; and water-soluble PEO and PVA, e.g., [22,23]. Filters from such polymers can be highly biodegradable, making the process and product environmentally friendly. 

Solution blow spinning also enables the production of fibres from solutions containing additives such as chitosan, ZnO, silver nanoparticles and essential oils, which impart additional properties to the fibres, such as bacteriostatic capabilities [24,25,26,27]. Chitosan on the fibre’s surface also enhances filtration efficiency by facilitating charge accumulation on the fibre surface [28].

Among the numerous advantages, solution blow spinning has its drawbacks. Its drawbacks include relatively low process efficiency (higher than electrospinning but lower than melt blowing) and scalability issues. However, using multiple nozzles can improve process efficiency, and further research on solution blow spinning can facilitate its scalability [15]. Therefore, this study aimed to analyse the process parameters of blowing PLA from a chloroform/acetone solution, leading to the design and production of multi-layer filters characterised by high filtration efficiency. We chose PLA because it is biodegradable, its use in polymer solution blowing is well described in the literature, and the fibres obtained from it are hydrophobic. They can be successfully used as filter material. Moreover, the PLA solution can be easily modified at the preparation stage to give the produced fibres the desired properties, e.g., bacteriostatic as a result of the presence of, for example, ozonated mormodica oil [29].

## 2. Materials and Methods

Poly (L-lactic acid) (PLA) (Ingeo™ 6202D, NatureWorks^®^ LLC, Minneapolis, MN, USA) solution was used in the polymer solution-blowing process. PLA was dissolved for 24 h on a magnetic stirrer in a chloroform (Sigma-Aldrich, Poznań, Poland)/acetone (Sigma-Aldrich, Poznań, Poland) (3:1) mixture; 6% and 10% (*w*/*w*) PLA solution were used in the tests.

### 2.1. Lattice Boltzmann Modelling 

**Figure 1:**
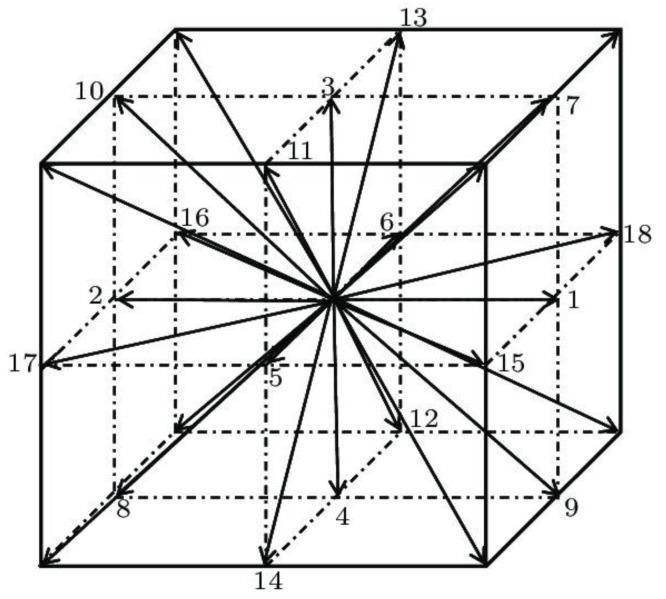
The cubic (3-dimensional) lattice with 19 allowed movement directions.

The lattice Boltzmann BGK approach modelled the fluid flow [30,31]. The cubic (3-dimensional) lattice with 19 allowed movement directions, usually referred to as D3Q19, was used (Figure 1). Aside from a particle at rest, the allowed directions are the middle of faces and edge. This approach was previously successfully used to model fluid flow in fibrous media [32].

The particle dynamics were determined using the Lagrangian Brownian dynamics algorithm [33]. The filter structure was approximated by a randomised staggered model with log-normal fibre size distribution described by Przekop and Jackiewicz-Zagórska [34]. The forthcoming fibre layers are normal to the neighbouring ones and are divided into identical cuboid unit cells. The diameter and position of fibre in the unit cell are chosen randomly. The size of the unit cell and the number of layers are calculated to obtain the desired thickness and porosity of the fibrous structure.

### 2.2. Solution-Blown Spinning

**Figure 2:**
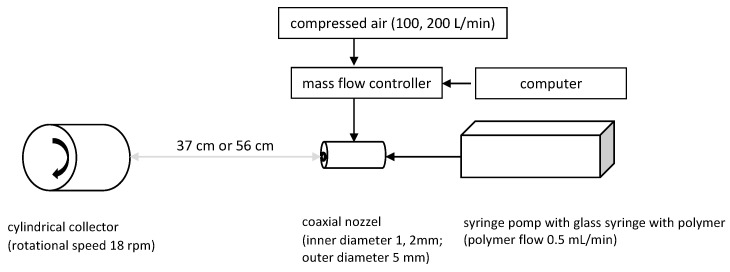
Experimental set-up.

The polymer solution-blowing installation consists of the following elements (Figure 2): a two-channel coaxial nozzle (internal channel diameter 1 mm or 2 mm, external channel diameter 5 mm), compressed air supply pipe, mass flow controller (SFC5500-200slm, Sensirion, Zurich, Switzerland) and the computer controlling it, syringe pump (Legato270, KDScientific, Holliston, MA, USA), glass syringe with a Teflon piston with a capacity of 50 mL (Hamilton, Reno, NV, USA) and a cylindrical collector (13 × 21 cm) arranged horizontally, covered by base fabric (21 × 41 cm) and rotating (18 rpm) along the long axis. A stepper motor provides the movement of the collector. The syringe pump with the nozzle was placed on a platform moving along the long side of the cylindrical collector. This allows even coverage of the entire collector surface with the generated fibres. A detailed description of the method of obtaining fibres in a polymer solution-blowing installation can be found elsewhere [35].

The polymer solution-blowing process was carried out using the parameters listed in Table 1. Fibre production was always carried out with a 0.5 mL/min polymer flow. Five filtration structures were manufactured for each set of parameters. For each sample, the mass of fibres deposited on it was determined as the difference in the mass of the fabric on the collector before and after blowing. The results presented in the article are average values from the five samples. Table 2 lists the analysed blowing variants. 

### 2.3. Analysis of Fibres Morphology

The morphology analysis of the obtained fibres was based on photos from a scanning electron microscope (TM-1000, Hitachi, Tokyo, Japan). Before examination, the fibres were sputtered with a layer of gold for 2 min (K550X EMITECH Quorum, East Sussex, UK). The average fibre diameter was determined based on the analysis of a minimum of 100 fibres taken from different areas of all samples produced for given process parameters. 

### 2.4. Filtration Efficiency

The studies conducted within this work were performed using the Palas MFP 2000 station (Palas GmbH, Karlsruhe, Germany). It was employed to analyse filtration in both initial and non-stationary states. Changes in the main parameters considered during the design of filtration materials, i.e., filtration efficiency and pressure drop, were examined both at the beginning of the process when the filter was clean and under actual working conditions during continuous loading with solid particles. In the experiments, a polydisperse silica-based dust named Arizona Fine Test Dust ISO 12103-1 (produced by Powder Technology Incorporated, Arden Hills, MN, USA) was used. It contains particles with diameters ranging from 0.26 to 16.60 μm, with the most numerous fraction consisting of particles with diameters of about 0.35–0.45 μm.

**Figure 3:**
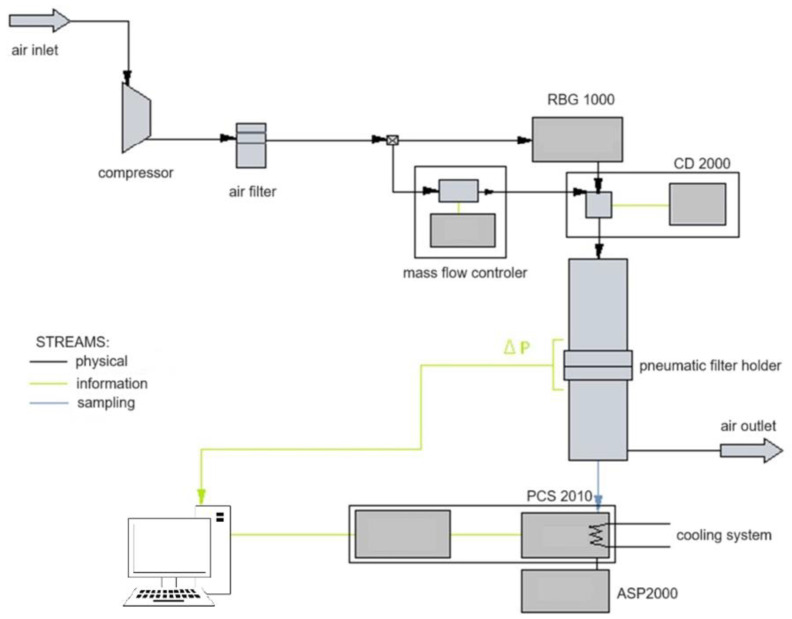
The test bench Palas MFP 2000 for the filtration process in flat materials.

The test bench comprised a piston-brush solid particle generator (RBG 1000, Palas GmbH, Karlsruhe, Germany), a pneumatic holder where the test filter was placed, an optical particle counter (PCS 2010, Katto Laboratory, Tokyo, Japan), a charge neutraliser (CD 2000, Palas GmbH, Karlsruhe, Germany) and a vacuum pump drawing steady aerosol samples for analysis (ASP 2000, Endress+Hauser, Reinach, Switzerland) (Figure 3).

For materials produced over a more extended period (those with a higher number of polymer fibres), the course of changes in filtration efficiency and pressure drops during the process duration was also determined.

## 3. Results

### 3.1. Mass of Deposited Fibres, Mean Fibre Diameter, Initial Filtration Efficiency and Initial Pressure Drop

**Figure 4:**
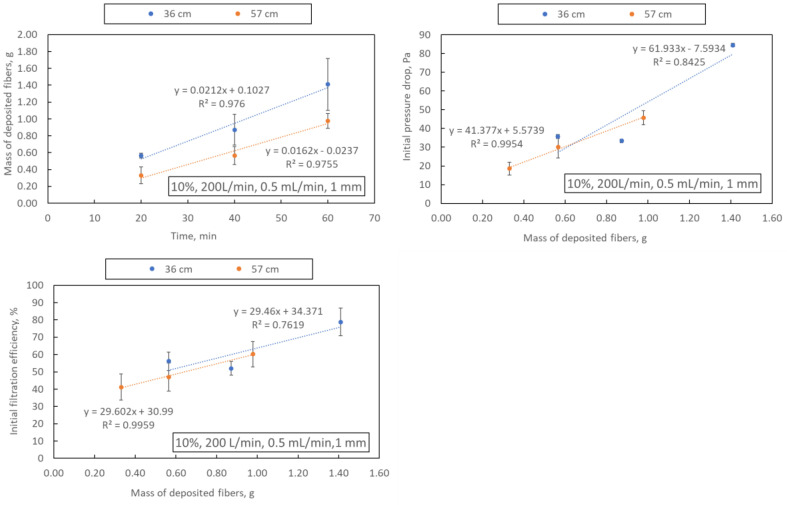
Dependence of the blowing time on the mass of deposited fibres and dependence of the mass of fibres on the filtration efficiency and pressure drop. The drawing shows the parameters of the process in the following order: polymer concentration, airflow, polymer flow and nozzle diameter.

**Figure 5:**
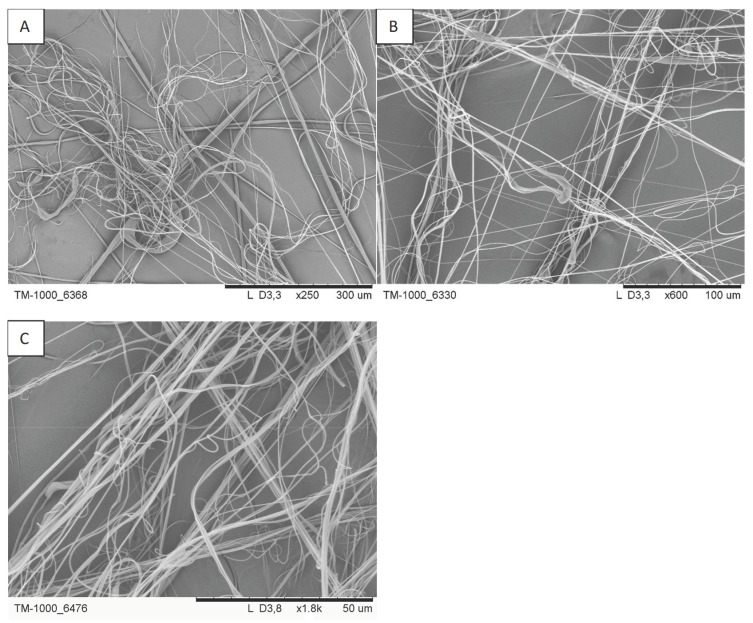
SEM photos of the selected filtration structures: (**A**) A6, (**B**) A7 and (**C**) A9.

The mass of deposited fibres, mean fibre diameter, initial filtration efficiency and initial pressure drop are shown in Table 3. As expected, extending the blowing time (samples A4, A8, A9 and A10–12) leads to an increase in the mass of fibres deposited on the collector (regardless of the distance from the nozzle to the collector, 36 or 57 cm), which also translates into an increase in the initial filtration efficiency and pressure drop (Figure 4). Examples of SEM photos of the obtained filtration structures are shown in Figure 5.

#### 3.1.1. Airflow

**Figure 6:**
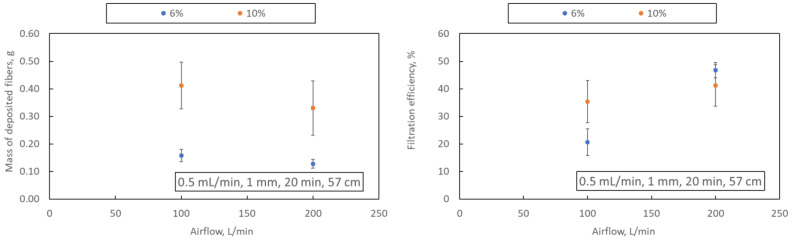
Dependence of the airflow rate on the mass of deposited fibres and filtration efficiency (samples A1, A2: blue dots; A3, A4: orange dots). The drawing shows the process parameters in the following order: polymer flow, nozzle diameter, blowing time and distance from the nozzle to the collector.

An increase in the airflow rate reduces the mass of fibres deposited on the collector (samples A1-A2 and A3-A4) when a 1 mm nozzle is used. This may result from generating a more significant number of fibres with a smaller diameter, whose capture efficiency on the collector is much lower and whose presence in the filter structure improves its filtration efficiency (Figure 6). The concentration of PLA does not change this effect. A significant increase in the mass of deposited fibres is observed when the airflow rate increases through a nozzle with a diameter of 2 mm (samples A6 and A7). This effect may be due to the relatively large diameter of the fibres obtained in this blowing variant (above 1 μm), the deposition efficiency of which is high.

#### 3.1.2. Nozzle Diameter

The influence of the nozzle diameter on the filtration process depends on the polymer solution concentration. For 10% PLA, an increase in the nozzle diameter causes an increase in fibre deposition on the collector, probably due to their growth in average diameter (samples A4, A7). It also increases filtration efficiency and a noticeable increase in pressure drop. In turn, for 6% PLA (samples A2, A5), an increase in the nozzle diameter causes an increase in fibre deposition on the collector, with a simultaneous slight decrease in their average diameter. It also causes a significant reduction in the initial filtration efficiency. This effect may be caused by too large a volume of solvent escaping from the nozzle per unit of time. As a result, a significant part of it does not evaporate, and no fibres are formed. The observed mass increase may also result from the deposition of polymer drops on the collector. Similar effects were observed by others [15].

#### 3.1.3. Polymer Concentration

The effect of polymer concentration on the average diameter of the obtained fibres (when using a 1 mm nozzle) is consistent with data from the literature [14]. As the polymer concentration increases, the solution viscosity and surface tension increase, which causes more excellent damping of the tensile and shear forces acting on the polymer stream at the nozzle outlet, which increases the diameter of the resulting fibres. This effect is visible for samples A2 and A4. An increase in the average fibre diameter following the classical filtration theory causes a decrease in filtration efficiency, which is also observed for samples A2 and A4.

All obtained structures are characterised by an initial filtration efficiency not exceeding 79% and a pressure drop not more significant than 84.5 Pa. To increase the filtration efficiency, the blowing time can be extended. This will increase the number of fibres in the structure, increasing filtration efficiency and pressure drop (as shown in Figure 3). Another way to increase filtration efficiency is to create a multi-layer filtration structure, the so-called sandwich structure, in which each subsequent layer would have greater filtration efficiency. Such a filtration structure could be produced in the blowing process from a polymer solution by changing the process parameters during blowing. 

The blowing parameters of samples A2, A8 and A10 were selected as optimal for producing a high-efficiency multi-layer filter. Podgórki, Bałazy and Gradoń [36] showed that even a small addition of nanofibers in the filtration structure significantly increases the filtration efficiency without causing a high increase in pressure. The tests showed that the smallest average fibre diameters were obtained when blowing with 6% PLA (samples A1, A2 and A5). The highest initial filtration efficiency of these samples was obtained for sample A2. Therefore, it was decided to use the blowing parameters of sample A2. In turn, the highest filtration efficiency of all samples was obtained for sample A10—therefore, the blowing parameters for sample A10 were chosen. The third blowing parameters (as for sample A8) were selected to increase filter lifetime and dust retention. An increase in the average fibre diameter causes a decrease in filtration efficiency but extends the dust loading. The blowing parameters for sample A8 enable the creation of a filtration structure with an average diameter of approximately 0.749 μm, efficiency higher than for sample A2 and a pressure drop of no more than 40 Pa. 

The lattice Boltzmann modelling was used to check whether the proposed assembly would have better filtration parameters than single filtration layers. Since the blowing time of the sandwich structure will be 120 min, two additional filtration structures (B1 and B2) were considered in lattice Boltzmann modelling. Structures B1 and B2 have an average fibre diameter similar to samples A10 and A2. Still, they are proportionally thicker to reflect the more significant number of fibres in the structure (due to the extension of the blowing time to 120 min).

Sample B3 is the composition of structures A2, A8 and A10, while sample B4 is the composition of A10, A8 and A2. In a multi-layer structure, the order in which the filter layers are positioned in relation to the filtered aerosol is essential. 

### 3.2. Lattice Boltzmann Modelling

The results of lattice Boltzmann modelling are summarised in Table 4. The study was carried out for the most penetrating particle size (MPPS)—300 nm. The structure of samples B1–B4 (porosity, thickness, average fibre diameter) used in the modelling is exemplary.

The modelling results obtained for single structures A8 and A10 are consistent with the experimental data. The filtration efficiency calculated for sample A2 is lower than the experimental one. As expected, the filtration efficiencies for the multi-layer structures are much higher than for the single filter layers (A2, A8, A10 and B2). However, they are lower than for a single B1 layer, which also has a high pressure drop, unfavourable in the filtration process. The filter structure approximation model had little impact on the obtained results. However, the modelling results did not confirm differences in the filtration efficiency and pressure drop of multi-layer structures depending on the order of assembly.

### 3.3. Multi-Layer Structure

Since the lattice Boltzmann modelling results confirmed the high filtration efficiency of multi-layer structures, four samples (B1–B4) were prepared using solution-blowing. The parameters at which the samples were obtained are listed in Table 5. The polymer flow was 0.5 mL/min (similar to samples A1–A12). Each of the B1–B4 samples was generated in five copies, and the results presented in the article are average results. 

Table 6 summarises the results obtained for the mass of fibres in each sample, the initial filtration efficiency and the initial pressure drop. The produced filtration structures are characterised by high (above 96%) initial filtration efficiencies (samples B1, B3 and B4) but also high (above 150 Pa) initial pressure drops (samples B1, B3 and B4). 

**Figure 7:**
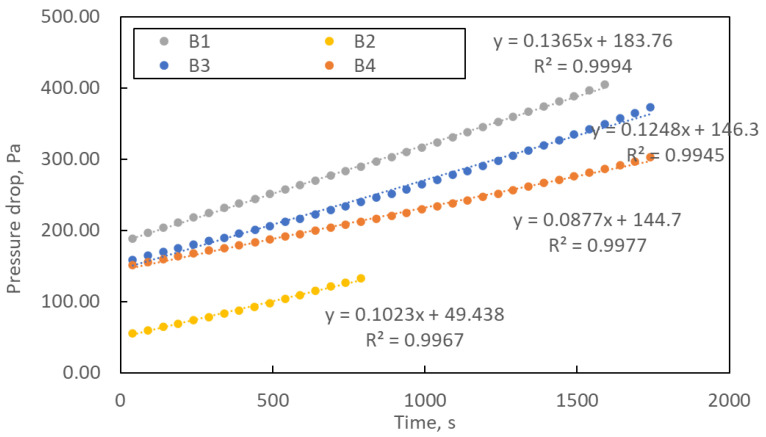
The study of loading samples B1–B4. Error bars are omitted for better readability.

Figure 7 shows the change in pressure drop during the loading process of the manufactured structures. The loading study was carried out until the pressure drop was three times higher than the initial one. The curves’ course shows that the pressure drops over time are linear. The exponential shape of the curve, characteristic of filter operation, was not achieved within the assumed loading time. The linear form of the curve means slow clogging of the filtration structures, which is a favourable phenomenon from the point of view of filter life. 

**Figure 8:**
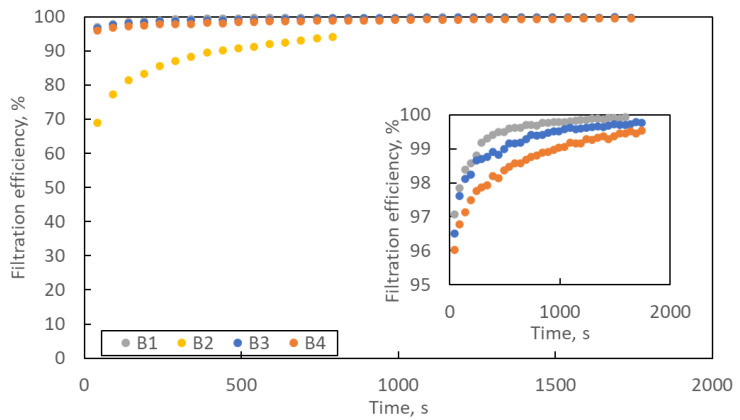
The changes in filtration efficiency of B1–B4 samples with loading time. Error bars are omitted for better readability.

Figure 8 shows the change in filtration efficiency with loading time. In the lower right corner is an enlargement of the Y axis in the efficiency range of 95–100%. The filtration efficiency increases with the loading time. At the end of the loading process, the filtration efficiency of samples B1, B3 and B4 exceeds 99.5%, and that of sample B2 exceeds 94%. 

## 4. Discussion

A perfect and universal filtration structure does not exist. Each filtration structure should be designed and selected for a specific purpose. Furthermore, the goal may be different. If we are looking for filtration structures that work for a long time and consume little energy, e.g., in air purifiers, the pressure drop across such a structure must be small. Still, the initial filtration efficiency will be low. Of course, it will increase as the filter is loaded and its porosity decreases, but it will never reach the values characteristic of HEPA or ULPA filters.

On the other hand, if we want high air purity, e.g., in life science, HEPA or ULPA, filters with high filtration efficiency will be the perfect solution. However, high filtration efficiency also entails much higher initial pressure drops and, consequently, higher operating costs. One of the determinants of the quality of the filter structure is quality factor. Quality factor makes it possible to link the two most important determinants of the filtration process: filtration efficiency and pressure drop.

Quality factor (*Qf*) is determined from the relation:

(1) $$Qf=\frac{-ln(1-E)}{∆p}$$

where *E* is filtration efficiency (-) and Δ*p* pressure drop across the filter (Pa).

**Figure 9:**
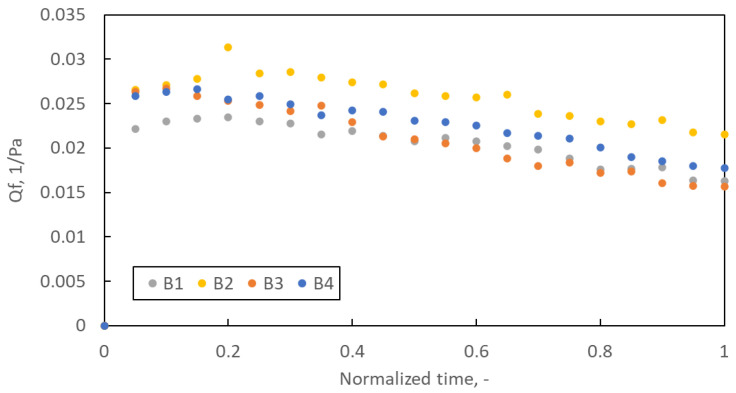
The quality factor as a function of normalised time.

Figure 9 shows the average quality factor for filter structures B1–B4 during the loading study. Due to the different lifetimes of each of the five samples prepared for each variant of the filtration structure, it was decided to present the Qf course as a function of the normalised time. The basis for time normalisation was the loading time of the tested sample.

**Figure 10:**
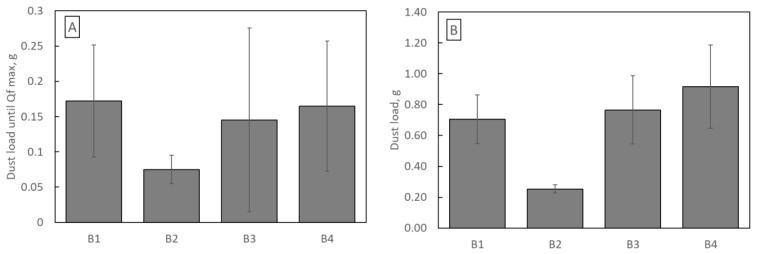
(**A**) The dust load until Qf reaches its maximum value; (**B**) total dust load.

The quality factors course is typical for the loading study. In the initial phase of filtration, Qf increases until it reaches a maximum, after which, due to the rapidly growing pressure drop and much slower increase in filtration efficiency, the Qf value decreases. Optimal use of filters should be at most until the maximum Qf is reached, but no longer than up to 2–3 times this time. Quality factor promotes low-pressure drop filtration structures. Therefore, for the B2 structure, Qf reaches the highest value. However, an essential parameter of filter operation is the amount of dust they can retain until Qf reaches its maximum value. Despite the highest Qf value, the B2 filter retains only 0.075 g of dust (the least of all samples B1–B4), while the B1 filter retains as much as 0.172 g (Figure 10A). 

On the other hand, sample B1 accumulated 0.70 g (per 100 cm^2^) of dust during the whole loading study. In comparison, sample B4 retained 0.92 g per 100 cm^2^ (Figure 10B). Equally important information as dust retention is the energy consumption of filtration structures.

**Figure 11:**
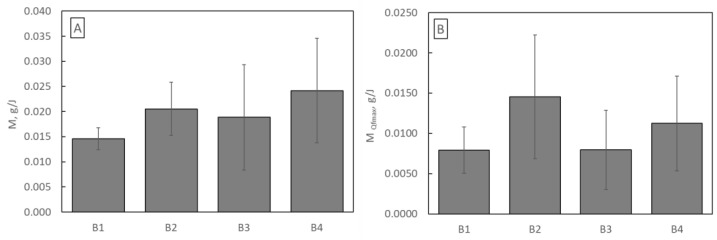
(**A**) Energy used to capture total dust; (**B**) energy used to capture dust until Qf reaches its maximum value.

By dividing the mass of dust deposited in the filtration structures by the power, according to the following relation:

(2) $$M=\frac{m}{t\Delta pQ}$$

where *m* is dust load (g), *t* is time (s), Δ*p* is pressure drop across the filter (Pa), and *Q* is airflow across the filter (m^3^/s), it was determined how much dust each sample can retain using 1 J of energy throughout its entire life (Figure 11A) and only until Qf reaches its maximum value (Figure 11B).

Considering the entire lifetime, the operation of the B4 filtration structure is the most energetically advantageous, while considering the lifetime until Qf reaches its maximum value, the operation of the B2 structure is the most energetically favourable. 

**Figure 12:**
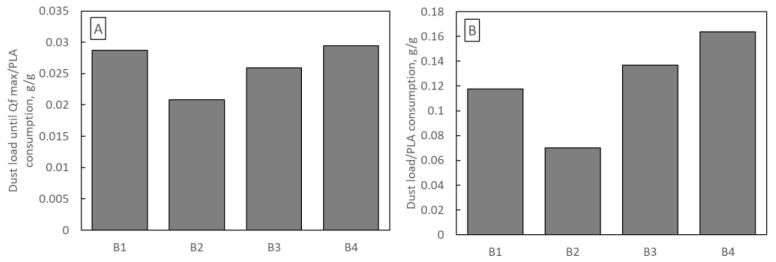
Mass of dust deposited on samples B1–B4 per 1 g of PLA used in production (**A**) until Qf reaches its maximum (**B**) total mass of dust.

An important economic aspect of filter selection is their production cost. Each of the B1–B4 samples was blown from a polymer solution for 120 min, so the difference in production costs results only from the difference in PLA consumption. Figure 12 shows the mass of dust deposited in samples B1–B4 per 1 g of PLA used in production. Figure 12A refers to the dust deposited until Qf reaches its maximum, and Figure 12B refers to the total mass of dust. The highest amount of dust (per 1 g of PLA) was deposited in sample B4 (0.029 g and 0.163 g) and the least in sample B2 (0.0208 g and 0.070 g).

Out of seven adopted criteria for assessing the produced filter structures, the B4 sandwich filter was the best in four. Despite comparable (with B1 and B3) initial filtration efficiencies and a similar pressure drop as B3, the B4 filter retains the highest amount of dust and has the lowest energy cost. Its production cost is also the lowest. However, looking at the quality factor, the B2 filter dominates with the lowest pressure drop and efficiency. The B2 single-layer filter retains the highest amount of dust per 1 J of energy until Qf reaches its maximum value. What is surprising is the much better result of the sandwich structure of B4 than B3. Lattice Boltzmann modelling did not indicate differences in both structures’ filtration efficiency and pressure drop. According to classical filtration theory, a multi-layer filter in which the efficiency of subsequent layers increases should be able to work longer, retain more dust and be more effective. However, the B4 filter, in which the first filtering layer has high efficiency, was better in practice. However, it is worth paying attention to the high error bar values.

Research has shown that the polymer solution-blowing process enables the creation of high-performance structures with the desired/designed filtration efficiency. It is possible due to the numerous parameters that control the process. Unfortunately, these multiple parameters are also responsible for high error bar values because polymer solution blowing is sensitive to even minor changes in process parameters. The problem of sensitivity to parameters will be even more visible when the scale of the process is increased, which is why further research on the polymer solution-blowing process and attempts to ensure stable, controlled conditions are fundamental.

## Figures and Tables

**Table 1:** The parameters of the solution-blowing process.

Parameters	Values	Units
internal channel diameter	1, 2	mm
time	20, 40, 60	min
PLA concentration	6, 10	% *w*/*w*
airflow	100, 200	L/min
distance from the nozzle to the collector	37, 56	cm

**Table 2:** Solution-blowing variants (A1–A12).

Sample No.	PLA Concentration, %	Time, Min	Internal Channel Diameter, mm	Airflow, L/min	Distance from the Nozzle to the Collector, cm
A1	6	20	1	100	56
A2	6	20	1	200	56
A3	10	20	1	100	56
A4	10	20	1	200	56
A5	6	20	2	200	56
A6	10	20	2	100	56
A7	10	20	2	200	56
A8	10	40	1	200	56
A9	10	60	1	200	56
A10	10	60	1	200	37
A11	10	20	1	200	37
A12	10	40	1	200	37

**Table 3:** Characteristic of the obtained filtration structures A1–A12.

Sample No.	Mass of Deposited Fibres, g			Fibre Diameter, μm	Initial Filtration Efficiency, %			Initial Pressure Drop, Pa		
	Mean	SD	CV, %	Mean	Mean	SD	CV, %	Mean	SD	CV, %
A1	0.16	0.02	14.43	0.365	20.61	4.84	23.49	8.18	2.42	29.63
A2	0.13	0.02	12.84	0.431	46.73	2.83	6.06	23.81	2.89	12.15
A3	0.41	0.08	20.62	1.330	35.33	7.63	21.60	13.31	3.82	28.72
A4	0.33	0.10	29.85	0.749	41.21	7.46	18.11	18.56	3.42	18.41
A5	0.17	0.03	16.52	0.396	25.76	3.56	13.82	9.77	1.43	14.64
A6	0.13	0.05	37.16	1.991	16.52	1.74	10.55	7.08	2.05	28.98
A7	0.45	0.13	28.63	1.241	49.37	11.23	22.74	24.57	6.78	27.58
A8	0.57	0.11	18.79	0.749	47.00	8.19	17.44	30.00	5.75	19.18
A9	0.98	0.09	9.01	0.749	60.20	7.37	12.25	45.66	3.72	8.14
A10	1.41	0.31	21.76	0.562	78.84	8.03	10.18	84.49	22.33	26.42
A11	0.56	0.03	4.79	0.562	56.10	5.22	9.31	35.65	2.64	7.42
A12	0.87	0.18	20.98	0.562	52.02	3.88	7.46	33.33	4.69	14.06

**Table 4:** The filtration efficiency and pressure drop of multi-layer filters and single-layer filters obtained by lattice Boltzmann. Monodisperse-regular, polydisperse-regular and polydisperse-staggered models approximated the filter structure.

Sample No.	Mean Fibres Diameter, µm	Standard Deviation, µm	Porosity, -	Thickness, mm	Monodisperse Regular		Polydisperse Regular		Polydisperse Staggered	
					Filtration Efficiency, %	Pressure Drop, Pa	Filtration Efficiency, %	Pressure Drop, Pa	Filtration Efficiency, %	Pressure Drop, Pa
A2	0.5	0.25	0.95	0.1	32.3	21.2	30.7	20.9	29.8	18.3
A8	0.8	0.4	0.9	0.2	52.6	38.3	49.2	37.8	48.6	35.2
A10	0.6	0.3	0.9	0.3	72.1	98.2	69.6	97.8	67.4	93.7
B1	0.6	0.3	0.9	0.5	93.2	217.3	91.4	214.2	89.9	208.6
B2	0.5	0.25	0.95	0.3	65.2	62.0	64.4	64.3	62.9	61.6
B3					92.1	157.7	89.3	156.5	88.2	147.2
B4					91.1	157.7	89.5	156.5	88.7	147.2

**Table 5:** Solution-blowing variants (B1–B4).

Sample No.	PLA Concentration, %	Time, min	Internal Channel Diameter, mm	Airflow, L/min	Distance from the Nozzle to the Collector, cm
B1	10	120	1	200	37
B2	6	120	1	200	56
B3	6,10	120	1	200	37–56
B4	6,10	120	1	200	37–56

**Table 6:** Characteristic of the obtained filtration structures B1–B4.

Sample No.	Mass of Deposited Fibres, g			Initial Filtration Efficiency, %			Initial Pressure Drop, Pa		
	Mean	SD	CV, %	Mean	SD	CV, %	Mean	SD	CV, %
B1	2.17	0.39	17.78	97.07	3.03	3.12	181.60	53.55	29.49
B2	0.62	0.03	4.18	68.85	9.43	13.70	50.80	11.26	22.16
B3	3.38	0.78	23.19	96.51	1.53	1.59	153.33	38.73	25.26
B4	3.38	0.78	23.19	96.29	2.04	2.12	155.20	46.75	30.12

## Data Availability

Data are contained within the article.
